# Time to be more efficient: reducing wasted transthoracic echocardiography (TTE) diagnostic appointment slots at Guy’s and St Thomas’ NHS Trust

**DOI:** 10.1136/bmjoq-2023-002317

**Published:** 2023-07-17

**Authors:** Dario Freitas, Sam Alner, Camelia Demetrescu, Grazia Antonacci, Nathan Proudlove

**Affiliations:** 1 Cardiology Department, Guy’s and St Thomas’ NHS Foundation Trust, London, UK; 2 Department of Primary Care and Public Health, NIHR ARC Northwest London, Imperial College London, London, UK; 3 Centre for Health Economics and Policy Innovation (CHEPI), Imperial College Business School, Imperial College London, London, UK; 4 Alliance Manchester Business School, The University of Manchester, Manchester, UK

**Keywords:** Coronary Disease, Diagnostic Services, Efficiency, Organizational, Statistical process control, Process mapping

## Abstract

Transthoracic echocardiography (TTE) is one of the most requested non-invasive cardiac imaging diagnostic modalities available in the National Health Service (NHS). There is persistently high demand, but nationally, activity has lagged, producing increasing numbers of breaches of the 6-week waiting time target. This delays patients’ diagnosis and treatment.

Patients attend hospital for TTE either as a clinic-linked or a standalone appointment. In this quality improvement project, we identified that the clinic-linked slots were a major source of wasted capacity due to both unbooked slots and a high rate of patients not attending their appointments (DNA).

DNA is a complex issue, aggravated in our trust by many IT systems, complex clinic-booking pathways and restricted patient communication channels. We parked changing these processes, pending an imminent, unifying IT development programme. We focused instead on unused clinic-linked appointments, with the goal of reducing these from 18% (~31 of ~175 allocated each week) to 5% by the end of the 14 week project period.

In close collaboration with service stakeholders, we identified that the primary root causes were related to the clinic-linked TTE booking pathway. The change idea was a 7-day rule: after reminders at 9 and 8 days prior to the clinic date, any appointment slots still unbooked by cardiology sub-specialities for patients attending clinic-linked appointments at 7 days, would be used for booking standalone TTE patients.

We refined this process over two plan-do-study-act (PDSA) cycles, reducing unused (wasted) appointment slots, allocated initially to clinic-linked patients, to a sustained level of 5.1%, meaning we could now perform approximately 21 additional TTE tests weekly; we have materially increased activity without increasing capacity.

This contributed to a significant reduction in 6-week TTE waiting-time breaches. Over the project, this went from 378 (30%, February 2022) to 71 (8%, September 2022) and latest data show 28 (4%, February 2023).

**WHAT IS ALREADY KNOWN ON THIS TOPIC**

- Demand for transthoracic echocardiography (TTE) has exceeded activity in the National Health Service (NHS), which has led to increased waiting times, delaying diagnosis and treatment and increasing breaches of the national 6-week waiting time limit target.

**WHAT THIS STUDY ADDS**

- Quality improvement (QI) methodologies have been applied in various healthcare settings to reduce waiting times and improve patient outcomes. However, to our knowledge, there is no previously published QI work in cardiology to improve booking pathways and increase TTE capacity.

**HOW THIS STUDY MIGHT AFFECT RESEARCH, PRACTICE OR POLICY**

- We demonstrate that fairly simple steps that improve booking administration can materially increase activity within the same resource capacity.

## Problem

Transthoracic echocardiography (TTE) is a diagnostic imaging technique that uses high-frequency ultrasound waves which are transmitted into the body; the reflected waves (echoes) are detected by a receiver and displayed on a computer monitor to show the internal structure and function of the heart. This provides detailed information about the structure of the heart chambers and valves and their overall size and function. It is a non-invasive and painless test (although the probe may need to be pressed relatively firmly onto the chest). There are no risks and the test can be safely performed on adults and children. Around 1.6 million echocardiography tests are conducted in National Health Service (NHS) England per year,^1^ almost all are TTEs.

Guy’s and St Thomas' NHS Foundation Trust (GSTT) is one of England’ largest NHS trusts, seeing 2.6 million patients annually across acute, specialist and community services in London’s Lambeth, Southwark and Lewisham boroughs.^2^

Prior to the COVID-19 pandemic, our cardiothoracic outpatient department saw around 65 000 patients and performed around 21 000 TTEs per year. TTE is, by far, the most requested cardiac imaging diagnostic—a high-volume service with the longest waiting lists in the cardiology specialty. Despite our efforts to reduce TTE diagnostic waiting times, the 6 months between October 2021 and March 2022 showed discouraging results, with a monthly average of 467 TTE breaches of the national 6-week waiting-time target. Along with persistently high demand for TTE, there have been chronic staff shortages that heightened the demand-capacity mismatch.

Our echocardiography team explored innovative processes, aimed at increasing diagnostic activity within the existent resources, focusing on maximising outpatient slot utilisation. We aimed to deliver service improvements without increasing staff work pressures or compromising their well-being. We considered reducing wasted slots the best ‘win-win-win’: (1) patients have shorter waits for diagnosis and treatment, (2) staff feel valued and not pressurised into working harder and faster to meet increasing demands and (3) the organisation meets targets without extra cost, while promoting good leadership practices and core trust values: ‘put patients first, be proud of what we do, respect others, strive to be the best, act with integrity’.^3^ Widespread staff dissatisfaction, increased work demands and stakeholders' need to improve the waiting list backlog were the motivators for pursuing this quality improvement (QI) work.

We used the Model for Improvement (MfI) framework that provides an integrated and systematic approach to QI, generating ideas driven by three questions: ‘What are we trying to accomplish?’, ‘How will we know a change is an improvement?’ and ‘What changes can we make that will result in improvement?’^4 5^ Resulting change ideas are then tested methodically through successive PDSA cycles, learning from the successes and failures in each cycle. The MfI has been used successfully in QI projects across hospital clinical sciences, including in other physiological sciences^6^ and in life sciences.^7–10^

Our project aimed to reduce the long patient waits for TTE (including breaches of the 6-week national target), by reducing the proportion of unbooked TTE appointment slots to 5% of the total allocation, by the end of the 14-week project period.

## Background

There is a high prevalence of cardiovascular disease (CVD) in the United Kingdom (UK), affecting around 4.0 million men and 3.6 million women each year, with associated annual estimated healthcare costs of £9 billion.^11^ Prevalence of coronary heart disease has remained constant at around 3% in England and 4% in Scotland, Wales and Northern Ireland.^12^ This results in around 64 000 premature deaths in the UK each year; an average of 175 deaths a day, or one every 8 mins.^11^ The NHS long-term plan recognises that CVD is the largest cause of premature mortality in impoverished areas and with the greatest potential over the next 10 years for the NHS to extend lifespans.^13^

Echocardiography is one of the most frequent non-invasive cardiac imaging diagnostic modalities in the NHS, essential for early detection and adequate management of CVD.^14–16^ This is similar in the USA.^17^

An independent review of diagnostics services for NHS England showed TTE demand grew by 5.7% per annum between 2014 and 2019.^16^ The review also highlights a growing shortfall of specialists, with trainee numbers lagging retirement rates.

In 2008, NHS England introduced a 6-week maximum diagnostic wait policy, pledging that only 1% of patients would wait longer than this.^16^ Prior to the pandemic, the NHS was already struggling to meet this target, with 7% of patient referrals for echocardiography in England exceeding 6 weeks (the number of cases on the waiting list longer than 6 weeks standing at about 5000); the pandemic then, of course, had a major impact and the breach rate has been around 45% since (at about 70 000 cases). National monthly data for echocardiography show activity rising slowly since 2021, waits potentially stabilising (at around 160 000 as of February 2023).^1^ Thus, the referral rate has long been greater than activity,^1 16 18 19^ suggesting a clear failure of policy and that current capacity and/or practices may be inadequate to achieve any target. Nevertheless, our department is striving to meet the NHS England guidance and our aim is to get down to under 5% of patients waiting 6 weeks or more by 2025.

QI methodologies have been applied in various healthcare settings to improve quality metrics and outcomes, especially in radiology.^20^ The literature contains echocardiography-related QI projects developing and implementing clinical practice. However, from a PubMed search for (QI AND increased TTE capacity), we found no publications that considered administrative practice and patient throughput.

As well as apparent lack of capacity, DNAs are a well-known major problem within the NHS, with a high financial and administrative burden on the whole healthcare system. Studies in the literature consider issues such as patients being unaware of their hospital appointment or forgetting it due to hospital failures in sending letters or text reminders and errors in the hospital’s patient database. They emphasise the value of addressing such internal issues before other solutions such as increasing capacity. Effective and efficient interventions include sending prompting letters and reminders such as text messages and emails.^21–23^

## Measurement

A TTE appointment slot is 45 min, and generally we have nine slots per day in each of around seven rooms (room numbers and allocation between clinic-linked and standalone varies). Overall, our data show that we have around 307 TTE appointment slots available per week.

Our initial baseline analysis considered all TTE slots available in the department over 2 weeks (31 January to 17 February 2022), with data extracted from our Cardiovascular Information Management System, which has been used to complete the TTE bookings. The figures in blue on the driver diagram (online supplemental figure S1) show the breakdown. We found 91 of 307 slots (30%) were wasted through being unbooked (vacant) or patient DNA. The problem was mainly the clinic-linked appointments, with around 65 of the 176 slots a week (37%) wasted; 36 (20%) through DNA and 29 (16%) through being unbooked. Therefore, we focused our QI project on these challenges.

10.1136/bmjoq-2023-002317.supp1 Supplementary data

The main cause, 55% of the wasted clinic-linked slots, was DNAs. The department allows a patient two DNAs before they are removed from the waiting list. The booking system has been set up to send letters and text-message reminders for all appointments. To investigate why we experience such high DNA rates, we conducted a survey. We phoned 83 DNA patients who had clinic-linked TTE appointments between 2 May and 30 May 2022. Of these 83 patients, 17 (20%) did not answer the phone, 20 (24%) reported not receiving any information about the appointment and 46 (55%) received the letter and text reminder but still DNA. Of these 46 patients, who received the letter and text reminder but still DNA, 30 (65%, 36% of the total 83 DNAs) said they did so because they forgot, despite all the information provided. The other 16 of these 46 patients (35%) could not attend for personal reasons but failed to call back to reschedule. Patients also mentioned that the text reminders were unclear as they did not mention that the appointment was for a TTE.

We feel strongly that all patient groups should be offered equal opportunities to access healthcare and there are many reasons why they may DNA. In view of the imminent trust digital technology upgrade, including a patient portal aimed at reducing DNA, we focused our project on reducing the unbooked slots (the other 45% of wasted clinic-linked slots). So, we defined metrics as follows: the outcome metrics (OM) are the unbooked slots allocated for clinic-linked TTEs, as both number of slots (OMn) and as a percentage of those allocated (OMp). The clinic-linked DNAs are process metrics (PMn and PMp), and the total number of TTE appointment slots allocated for clinic-linked booking is a balancing metric (BM).

**Figure 1:**
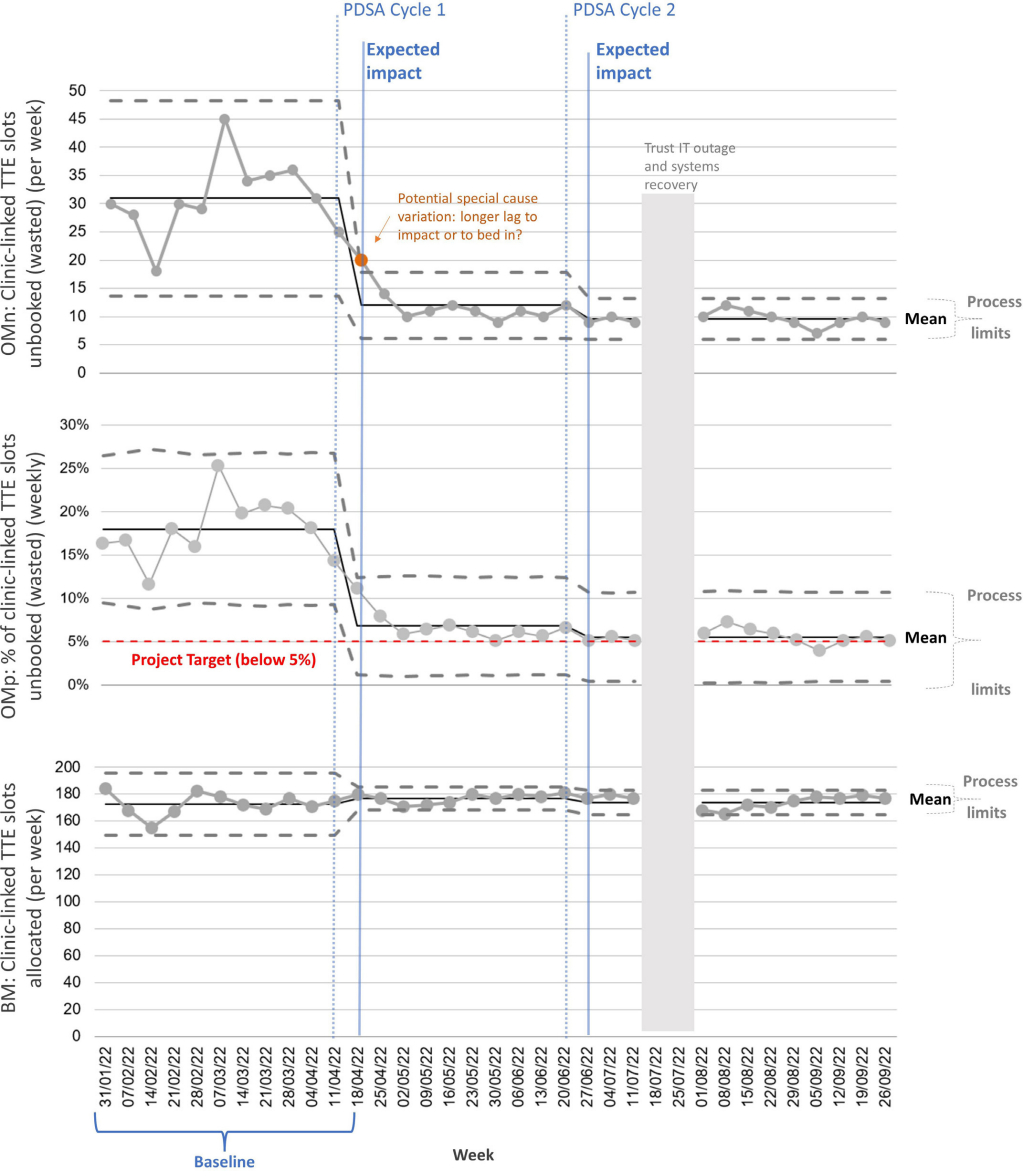
Metrics over time. BM, balancing metric; OM, outcome metric; PDSA, plan-do-study-act cycle; TTE, transthoracic echocardiography.

Online supplemental figure S2 shows OMp, PMp and BM daily for the 2-week initial snapshot, with our internal targets (aspirations) for waste, due to each source, of <5%. The percentage data are volatile over such short time intervals but show no strong day-of-week pattern. Figure 1 shows our focus: the unbooked slots (OMn and OMp), along with the total available (BM), with weekly aggregation over 11 weeks, to give more reliable baseline estimates. We used the NHS statistical process control (SPC) chart templates.^24^ The graphs show the means and process behaviour limits, as the expected range of random variation.^25^ Our goal, to reduce the waste of capacity due to unbooked slots to 5%, is also indicated. In the baseline period, the process is statistically stable (exhibiting only random variation), but with high process variation. The baseline mean is 31 slots unbooked per week (OMn), this being 18.0% (OMp). Slots available (allocated for clinic-linked TTEs) (BM) are very stable across the period of this project, at about 175 per week.

**Figure 2:**
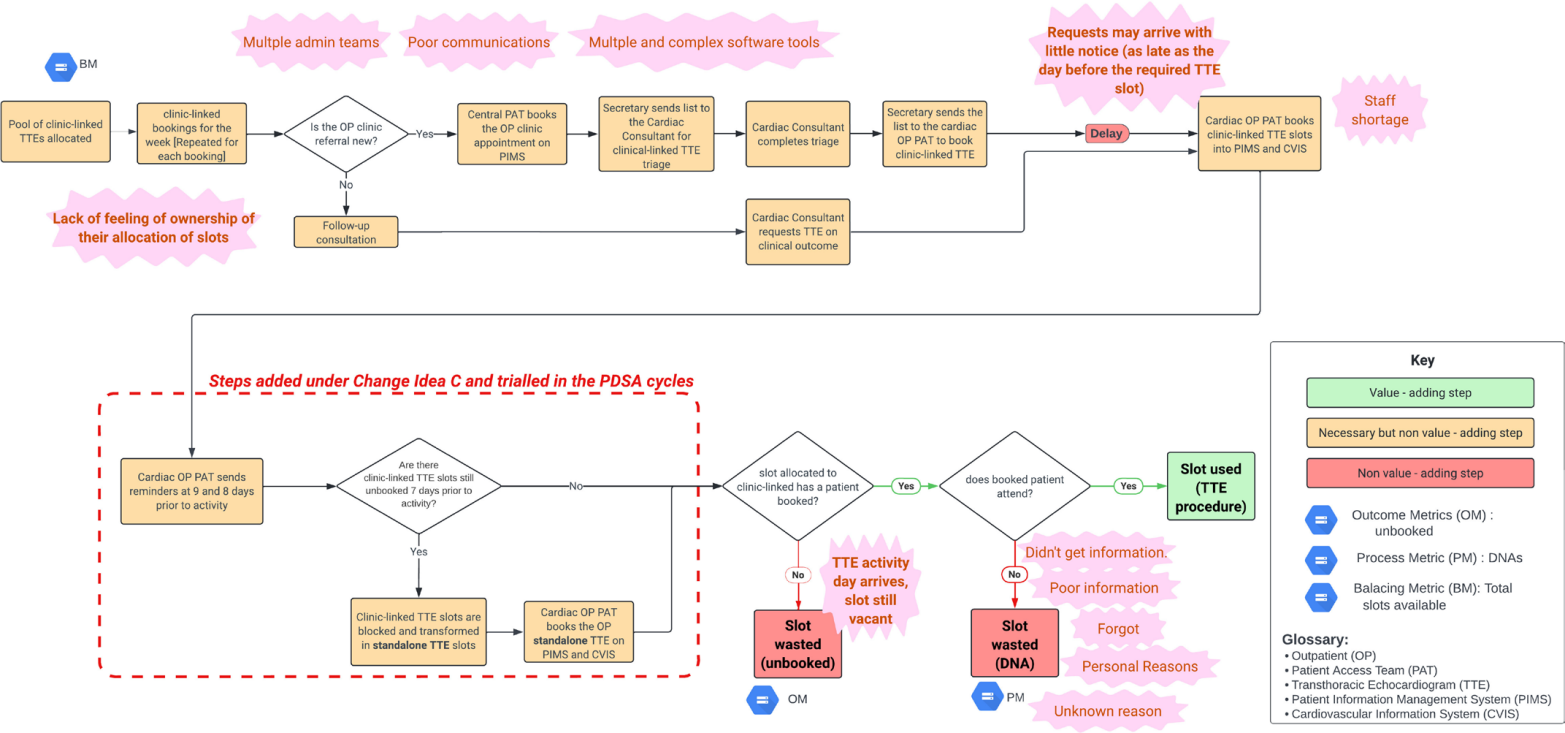
TTE service process map. PDSA, plan-do-study-act cycle; TTE, transthoracic echocardiography.

It was essential to understand and identify potential sources of waste in the current booking workflow. We mapped the process with service stakeholders^4 26 27^ (see figure 2), highlighting a significant booking delay. Sometimes TTE referral requests arrived at the cardiac outpatients’ patient access team (PAT) as late as the day before the specialist clinic (and so the required linked TTE). Therefore, as a consequence, a material number of clinic-linked TTE slots were unused, creating ‘waste’.

We conducted root cause analysis using a fishbone cause-effect diagram^4^ (see online supplemental figure S3). Persistent shortage of administrative staff and the involvement of different teams to finalise the booking pathway led to poor communication. In addition, the information technology (IT) booking system has been very complex, with several different software tools required. The lack of ownership taken by the various cardiology subspecialities in making use of the pool of clinic-linked TTE slots led to waste. All these factors contributed to the overall waste identified (see figure 2).

## Design

The magnitude of the unused capacity required a prompt solution and it was essential to involve stakeholders to reach consensus. We set up a QI team comprising of stakeholders from different groups, including the echocardiography manager, the outpatient cardiac diagnostic service manager, the cardiovascular directorate assistant service manager and the cardiology consultant team. After reviewing the issues identified in the process map, the driver diagram and root cause analysis (see figure 2, online supplemental figure S1-S3), the QI team initially identified four potential sets of ideas for change:

- Creation of a dedicated administrative team for clinic-linked TTE booking.
- Upgrading the IT booking system.
- Introducing a ‘7-day rule’ allowing transfer of unbooked clinic-linked TTE slots to standalone TTE booking.
- Changing the clinic-linked TTE appointment letter template to state a single arrival time allowing for the preclinic TTE, instead of sending two separate letters that often confused patients.

The driver diagram (online supplemental figure S1) shows how we expected each of these change ideas to impact on our system.

Idea A was rejected by cardiothoracic outpatient management due to current financial constraints and on-going staff shortage challenges.

Idea B had to be delayed as the trust has been planning the implementation of a new electronic patient record (EPR) system (EPIC software) in 2023. This will replace the current fragmented systems, and it is expected to markedly improve booking pathways and processes.

Idea D was initially considered as a feasible step towards addressing the DNA problem. However, on closer investigation, we found that this involved changes in the clinical consultation codes used to complete bookings on multiple IT systems. This would have been a very complex and lengthy process, so was postponed pending implementation of the EPIC EPR.

Idea C was, therefore, the focus for our project. We realised that many slots allocated to the various cardiology subspecialities for same-day preclinic TTEs were not utilised due to poor coordination, late bookings and delays in communication.

We know that when capacity is split up inflexibly into separate pools (here between clinic-linked and standalone TTEs), it is ‘carved out’; when demand is variable, the rigid demarcation reduces the total use of capacity (activity).^28^ In our system, slots were set aside for the linked clinics, but if not required, there was no mechanism to allow release of these slots to the separate waiting list of standalone TTE patients.

To reduce this carve-out waste, we proposed a ‘7-day rule’: at the point 7 days before the clinic date, any of the clinic-linked TTE slot allocation that remained unbooked, would be transferred to, and booked as, standalone TTE. The cardiology subspecialities became responsible for communicating their bookings before this 7-day cut-off. The stakeholders agreed with the QI team that this would be acceptable if the cardiac PAT sends a reminder to the subspecialty teams about the upcoming TTE clinic-linked slots 9 and 8 days prior to the clinic.

In reality, there will always be some unbooked clinic-linked slots, for example, due to short-notice cancellations, etc, and our project’s target was to get this down to 5% (corresponding to between 6 and 13 clinic-linked slots still unbooked). If this headroom was to be exhausted and there were further urgent cardiologist requests, we can schedule the patient into a standalone slot (and the patient might then have a telephone follow-up) and admin teams would rearrange booked slots, so urgent patient care would not be compromised. We discussed this policy with the cardiac outpatient service managers, and all stakeholders agreed that this seemed a sensible idea to trial.

## Strategy

To test and refine the 7-day rule, we conducted two PDSA cycles (see table 1).

**Table 1:** Improvement PDSA cycles

PDSA cycle	Plan/prediction	Do	Study	Act	Time required
Baseline	Go-and-See	Analyse data and set up baselines in SPC templates	OMn=31.0 unbooked slots/week OMp=18.0**%** of allocated BM=172.55 slots allocated/week	A very material waste of capacity: experiment with feasible change ideas	11 weeks
PDSA cycle 1	**Implement 7-day rule** Transferring clinic-linked TTE booking ownership to the different cardiology sub-specialities.	Cardiac OP PAT team agreed would send a reminder about the TTE clinic-linked slot 10 days prior to the activity. If no reply 7 days prior to activity, these would be blocked-out by the cardiac OP service manager and transformed into standalone TTE slots in a way to guarantee the TTE booking optimisation. Use a questionnaire survey to gather staff feelings.	OMn=12.0 unbooked slots/week OMp=6.8% of allocated BM=177.0 slots allocated/week (no material change) Positive staff responses on need and value of change; some admin unease about support.	Very material reduction in waste of slots that had been allocated for clinic-linked TTEs. Adopt as permanent change. BUT reassure admin staff on support, involve service managers team	10 weeks
PDSA cycle 2	**Reinforce the 7-day rule**	Meet with all admin team, Q&A. Present QI project methodology, its results and the staff survey feedback during the monthly cardiac administrative team meeting. Create an opportunity to engage directly with the team, get more feedback on the change idea in the present, and consider future actions by service managers to fulfil team requests.	OMn=9.6 unbooked slots/week OMp=5.5% of allocated BM=173.9 slots allocated/week (no material change)	Further worthwhile improvement observed and sustained. Adopt as a refinement.	14 weeks (but IT failure during 2 weeks: 12 weeks of data)

BM, balancing metric; OM, outcome metric; PDSA, plan-do-study-act cycle; SPC, statistical process control; TTE, transthoracic echocardiography.

### PDSA cycle 1

The 7-day rule was trialed. The additional steps are indicated in figure 2. After the reminders, if 7 days prior to the day of the activity the administrative teams had not booked, or communicated the need to book, all their slots then these would be blocked out by the cardiac outpatients’ service manager and transformed into standalone TTE slots. We predicted that this would reduce unbooked clinic-linked slots, contributing to overall utilisation of TTE capacity.

Figure 1 and table 1 show the results. Note: because of the expected time lag between introducing the rule and its potential impact on waste (7 days later after the transfer of unbooked slots), the performance regime is taken to (potentially) change a week later than the start of a PDSA cycle. We see a considerable step-change improvement. The mean unbooked slots (OMn) drop from 31.0 to 12.0 per week (18% to 7%, OMp), and with much lower variation. Consequently, the rule was adopted as a permanent change, but we were still short of our target (5%).

The changes in OMn and OMp suggest that the impact may possibly have taken more than the 1 week we have allowed for, with a ‘special cause’ (possible non-random behaviour) warning on OMn (the orange data point, though it is only just over the upper process limit). This was in an environment where the slots allocated (BM) remained almost identical, though more consistent.

The change did, however, materially impact the cardiac administrative team’s daily practice with additional steps added to their processes (see figure 2), requiring more admin time. Following good change management practice, we consulted those affected and gathered their opinions during the change. Given the size of the overall team involved, we used an online survey with eight questions and an estimated completion time of 2 min. We disseminated it during PDSA 1 and left it open for 2 weeks (23 May to 6 June 2022). It was sent to 30 members of staff, including administrative staff involved in booking, the service managers and clinical leads. There were 13 replies (43% response rate), the majority from the administrative staff (5 replies, 38% of respondents). Questions about their understanding of the reasons for the change, and their opinion on whether this would make the booking process more efficient, received positive responses from 70% of respondents. However, questions about getting support before, during and after implementation of the change received mixed responses. A summary of themes elicited by the survey is summarised in a 4N chart^29^ in online supplemental figure S4.

### PDSA cycle 2

Therefore, we attempted a second cycle with reinforcement of the change process and further engagement, particularly with the administrative staff. To support the project, we presented the QI project methodology, its results and the survey feedback during the monthly meeting of the cardiac administrative team, coordinated by the cardiovascular service manager, on 22 June. That created an opportunity to engage directly with the team, get additional feedback on the change plan and discuss future actions required to address general administrative concerns, as highlighted in the 4N chart (online supplemental figure S4).

The results show a further, though small, improvement, from a mean unbooked slots level (OMn) of 12.0 to 9.6 per week (7% to 5.5%, OMp)—see figure 1. The SPC chart indicates that we can sustainably achieve our 5% target in nearly half of weeks in future. There are two data points (weeks) missing from the SPC. In mid-July 2022, the UK experienced an extreme heatwave, leading to both the Trust’s IT datacentres shutting down, resulting in cancellations of procedures and staff resorting to paper records; the impact was considerable and took weeks to fully recover.^30 31^

## Results

The overall changes are shown in the SPC charts (figure 1). We see an overall sustained reduction from a mean of 31.0 to 9.6 appointments lost each week due to being unbooked for clinic-linked patients, from 18.0% to 5.5% of their weekly allocation. Further improvements would be needed to reliably achieve the under 5% weekly target.

We have reduced waste by up to 21 TTE slots each week so, an increase in potential activity of over 1000 procedures per year, 7.5% of our total capacity no longer wasted. We can perform more TTEs—both clinic-linked slots through better and more prompt booking, and standalone TTEs through the ‘7-day rule’ release of unbooked clinic-linked slots. This contributed to our reduction in TTE 6-week target breaches.

NHS England data spreadsheets for the situation at month ends, contain echocardiography wait data at provider (eg, trust) level. This aggregates nine Operating Procedure Supplement Codes (OPCS),^32^ but in our internal GSTT data around 94% of the echocardiography waiting list has been TTE (OPCS U20.1). We present here extracts from the NHS England data for our trust versus the aggregate national data, in the format:

- patients who have waited more than 6 weeks/total waiting list = % breeches.

As of end of February 2022 (just before the start of our changes):

- GSTT: 378/1275 = 30% versus nationally: 68 241/163 734 = 42%.

As of end of September 2022 (end our QI project reported in this paper):

- GSTT: 71/881 = 8% versus nationally: 76 291/159 474 = 48%.

As of end of February 2023 (latest data):

- GSTT: 28/726 = 4% versus nationally: 61 647/161 288 = 38%.

The public data do not contain referral numbers, but the aggregated waiting list across the 15 diagnostic tests in the reporting system (1.6 million patients at the end of February 2023) has continued a strong upward trend since 2008, making sustaining any waiting-time target infeasible. The national 1% 6-week breach rate was last achieved in late 2013 and continued creeping up until the shock of COVID-19, when it reached nearly 60% in mid 2020. It came down to around 20% in mid 2021 but has been increasing again since, standing at 25% as of the end of February 2023.^1 33^

For England as a whole, the echocardiography waiting list is the fourth largest of the 15 and has the third highest number of 6-week breaches, which is the joint highest proportion.^1^ The data above and in summary analyses^33^ suggest that echocardiography may be one of the few diagnostic test areas to have stabilised, though there is little (at best very slow) progress on reducing waits.

At GSTT, we are clearly doing very much better than England as a whole, both in reducing our waiting list and the proportion of patients waiting over 6 weeks. We have achieved the Trust’s <5% 6-week breach target well before the 2025 horizon, and the latest internal data suggest we may soon reach 1%. From our monthly meetings, staff engagement has remained strongly positive.

## Lessons and limitations

The most significant limitation in our QI project was the inability to test three out of four change ideas in the prevailing Trust circumstances, but we are planning to revisit them later in the year, after the major IT implementation of the new EPR (EPIC) and IntelliSpace Cardiovascular IT system. The TTE workflow (figure 2) is fairly straightforward relative to many other complex hospital pathways, so there are limited windows for improvement.

Action is clearly needed to address the DNA waste. Our analysis found 20% of clinic-linked and 16% of standalone TTE slots were wasted through DNA, around 57 slots per week in total (nearly 3000 per year). Although our change plan seemed straightforward, we realised it required less-than-straightforward changes to the already-overstretched IT booking systems, staff working patterns and close collaborative work across admin, clinical and managerial teams. However, we are considering the launch of a new QI project after the Trust IT upgrades and EPIC implementation.

As mentioned earlier, we feel strongly that all patients should be offered equal opportunities to access healthcare and there are many complex reasons why they DNA. We understand that this is inevitably dependent on human behaviour, particularly when patients are outside the healthcare environment. However, we will focus on working with IT in developing new digital patient portals that should facilitate more flexible patient access to hospital care.

Root causes for waste from unbooked clinic-linked slots were easily highlighted with straightforward QI tools. The main issues identified were in the booking process ‘upstream’, related to poor communication channels within different teams and across the hierarchy in cardiology. Our ‘7-day rule’ change project involved the implementation of additional steps downstream, our main area of influence, to reduce the impact on capacity usage. In QI, we usually try to eliminate steps and avoid workarounds, which tend to accumulate in healthcare processes,^32^ but sometimes we do have to add workaround steps to address the problems arising from areas outside of our direct control and influence. We note that it would have been preferable to change habitual behaviour in the upstream booking steps and change the IT systems, but these options will require a much longer implementation time. However, the new reminders, in our case from cardiac outpatients PAT, are promoting a habitual change upstream, as we continue to receive more and earlier clinic-linked bookings.

A general observation from this project concerns the perception and attitude of stakeholders to change. Within the various physiological clinical science areas across the NHS, issues related to waiting lists, wasted capacity and poor IT infrastructure are well-known. In our department, these had been identified by stakeholders previously, but despite awareness of the negative impact of all these problems on service workflows, there was initial resistance to conducting QI. We experienced similar limitations and inertia on ability to access useful data. While most data were relatively accessible, some indicators, such as the total number of TTE breaches, were only available monthly instead of weekly, which would have been useful for this project. Other data were hard to collect or time intensive to analyse, for example, use of clinic-linked slots had to be observed and recorded manually.

These phenomena might indicate an organisational culture, which would benefit from a refreshed vision on openness to change and better use of operational data within a quality management system.^34 35^ As common, in retrospect, it would have been useful to have several more process metrics, for example, to detect changes in activity in the clinic-linked and TTE streams.

Another obstacle we encountered was the department’s shortage of administrative staff and insufficient acknowledgement by very senior management of its critical negative impact on clinical services delivery. Despite the positive results obtained in PDSA cycle 1, the administrative team faced on-going staff shortages, and the lack of human resources was an insurmountable barrier to creating a unified booking team (change idea A). There was also an understandable associated degree of dissatisfaction among the admin team, as captured in the 4N chart (online supplemental figure S4). It is notable that the cost of additional administrative staff recruitment is significantly below the (nominal) cost of the lost clinical activity, nevertheless the achievement of a joined-up cost-benefit philosophy continues to be a long-term struggle within the complex NHS system. In all business plans, service managers have the duty to emphasise the consequences of failing to sustain adequate staffing levels, including the administrative support teams that enable clinical services to function efficiently.

As a learning point, we should have asked stakeholders to complete individual 4N charts, which would have allowed comparison with the QI team’s views and investigation of alignment of the project vision within the organisational hierarchy.

The stable total number of clinic-linked slots available (BM in figure 1) show that our results were not confounded by changes in expected workload (and staffing) or a change in allocation. The period of major IT outage in July also appears not to have had a marked knock-on effect.

The final result of this change process is the considerable improvement in the utilisation of pre-existent capacity that was previously poorly utilised. It is an example of reducing the waste caused by capacity ‘carve-out’.^28^ The project allowed us to meet the target waste level of less than 5% waste of the clinic-linked appointment allocation due to unbooked slots, most of the time (figure 1). While this is a major improvement and was a useful ‘stretch’ target, the data suggest that it is very ambitious to expect to implement further similar changes to achieve 5% reliably, particularly given the remaining variation in the system evident in the wide process behaviour limits.

## Conclusion

Echocardiography is a very important non-invasive cardiac imaging diagnostic tool, widely used in the NHS. The imbalance between demand and ‘capacity’ is well known and, despite efforts to increase capacity, actual activity is still far from desired at the national level.

We found QI methods such as the MfI very useful in investigating capacity-demand problems in our trust. They highlighted that, in practice, our activity (here, TTE procedures delivered) was far below the (theoretical) capacity, helped us identify the main causative problems and prompted disciplined experiments aimed at delivering significant improvements in activity.

The project reveals that it is critical to understand barriers when implementing any change process in highly complex managed systems such as the NHS. Timeliness and clarity of communication flow between teams, and the availability of resources (human, infrastructure, IT, etc) is commonly less than would be required for highly efficient patient flows, adding pressure on the system and staff. Engagement between the many stakeholders is imperative to foster the willingness of already overworked NHS staff to commit to QI.

This QI project demonstrated that the waste of capacity related to clinic-linked TTE bookings could be addressed by implementing additional steps, effectively creating extra capacity, without additional resources. More generally, this provides an example of tackling waste of clinical capacity by adding ‘fixes’ to reduce the consequences of inefficient and fragmented booking systems in the NHS.

## Data Availability

All data relevant to the study are included in the article or uploaded as supplementary information.
