# Supplementary material for: Time to be more efficient: reducing wasted transthoracic echocardiography (TTE) diagnostic appointment slots at Guy’s and St Thomas’ NHS Trust
Source: BMJ Open Qual. 2023 Jul 17;12(3):e002317. doi: 10.1136/bmjoq-2023-002317 (PMC10357679; doi:10.1136/bmjoq-2023-002317)
Supplement: Supplementary data [file bmjoq-2023-002317supp001.pdf]

Figure S1: Driver Diagram

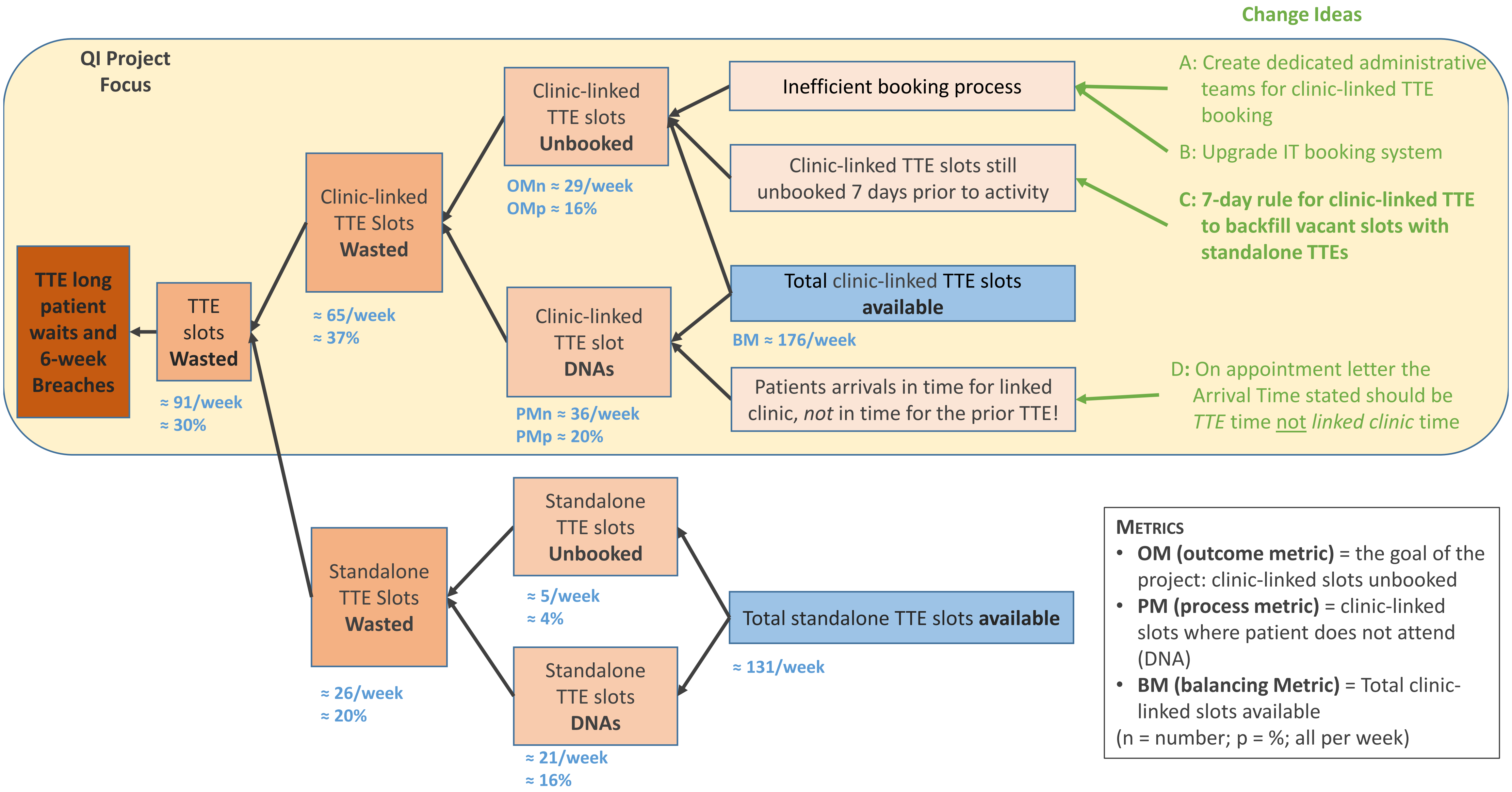

BMJ Publishing Group Limited (BMJ) disclaims all liability and responsibility arising from any reliance placed on this supplemental material which has been supplied by the author(s)

Figure S2: 2-Week Initial Snapshot of Daily Clinic-Linked TTE Appointment Slots

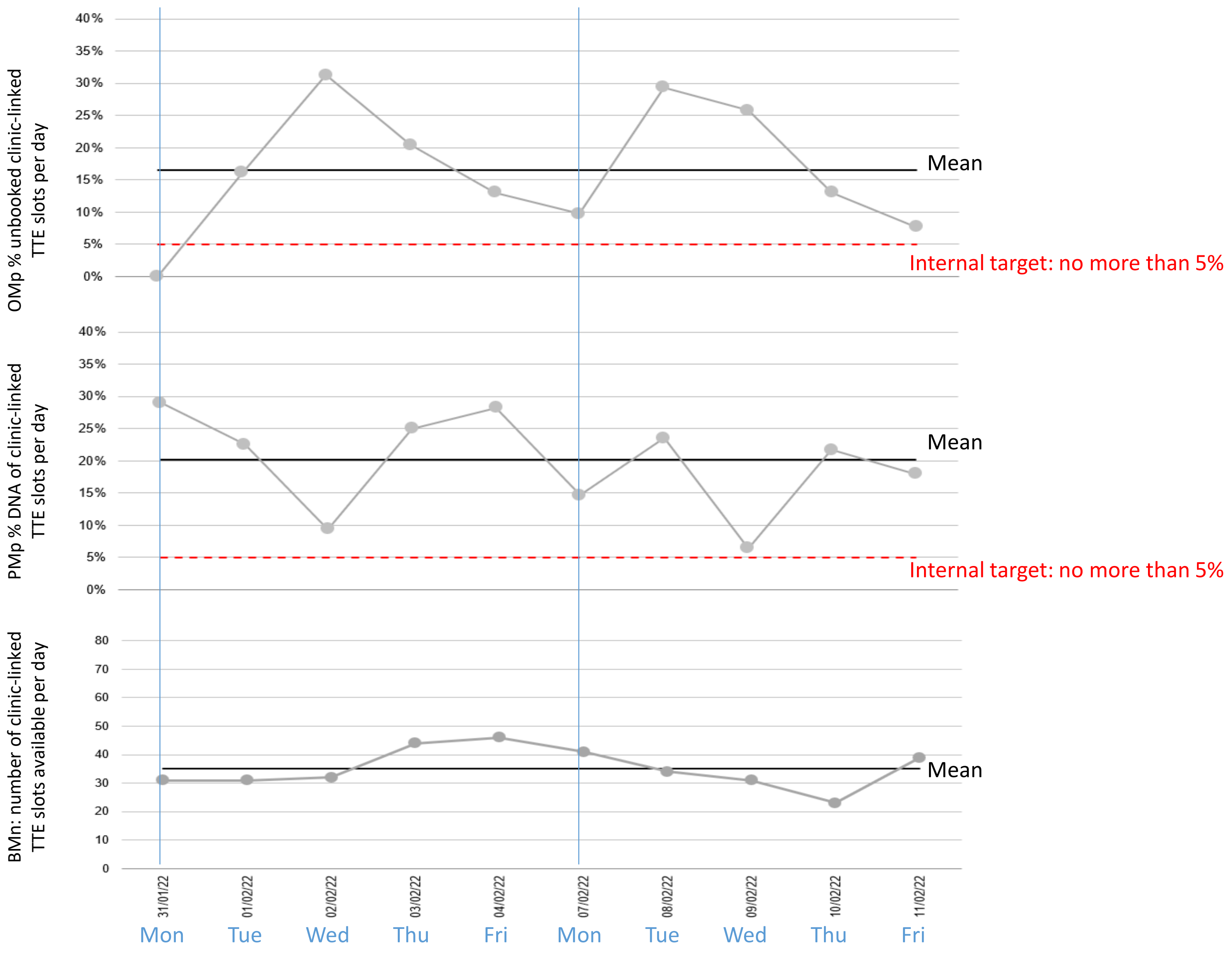

Figure S3: Root Cause Analysis (Fishbbone) Diagram

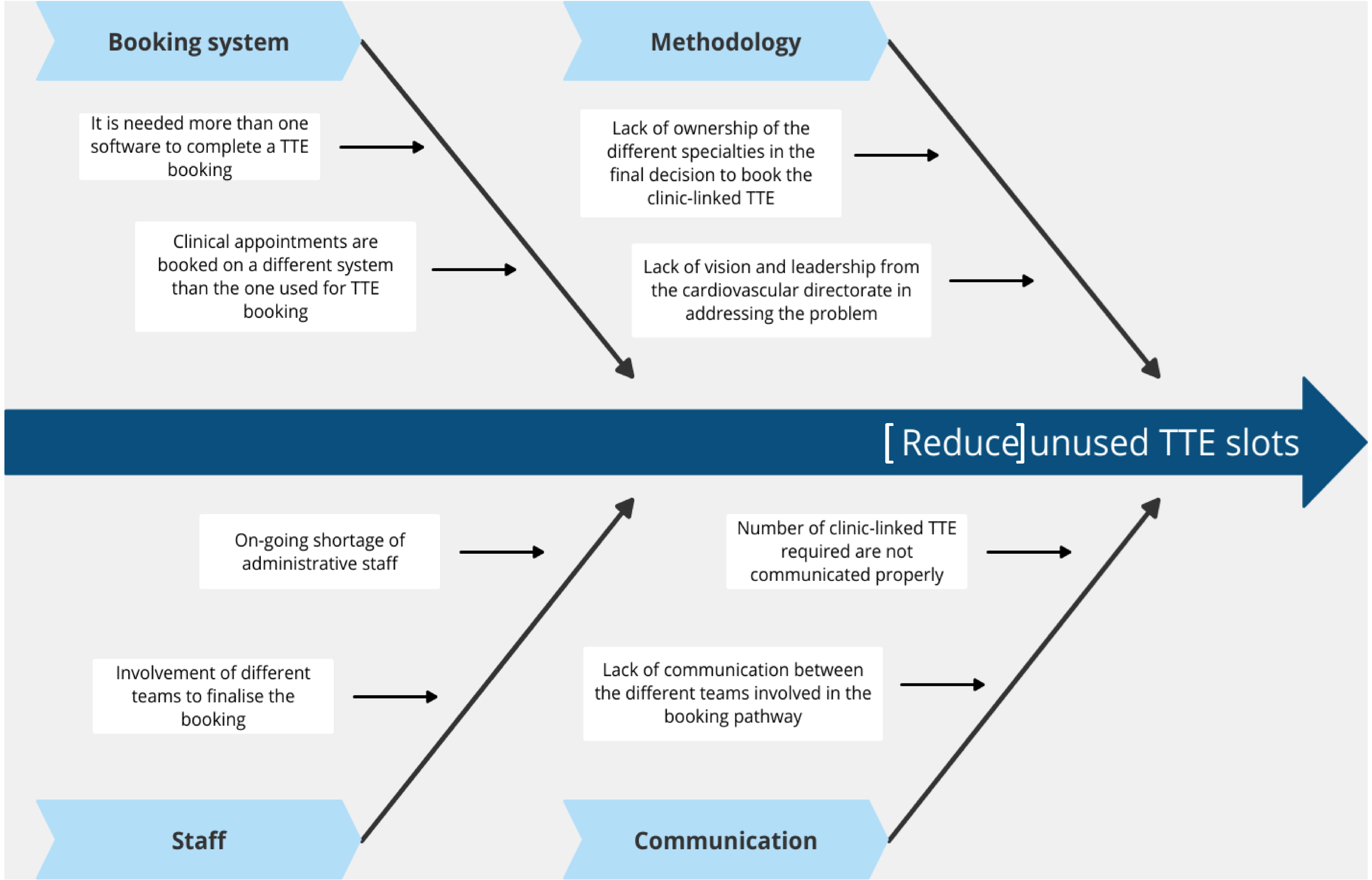

BMJ Publishing Group Limited (BMJ) disclaims all liability and responsibility arising from any reliance placed on this supplemental material which has been supplied by the author(s)

Figure S4: 4Ns Chart

	Present	Future
	**Nuggets**	**Nice Ifs**
+ve	Professional team<br>Recognition of current process failures<br>Staff willing to try change	Staff feel valued<br>Adopt sustainable changes<br>Consistent support from administrative teams
	**Niggles**	**No Nos**
-ve	Poor communication of changes<br>Difficult to manage workload<br>Poor IT system to process bookings	Feel overwhelmed with workload<br>Lack of support from the service managers<br>Not promoting well-being in the workplace
